# Biocompatibility and Cytotoxicity of Gold Nanoparticles: Recent Advances in Methodologies and Regulations

**DOI:** 10.3390/ijms222010952

**Published:** 2021-10-11

**Authors:** Małgorzata Kus-Liśkiewicz, Patrick Fickers, Imen Ben Tahar

**Affiliations:** 1 Department of Biotechnology, Institute of Biology and Biotechnology, College of Natural Sciences, University of Rzeszow, Pigonia 1, 35-310 Rzeszow, Poland; 2 TERRA Research and Teaching Centre, Microbial Processes and Interactions Laboratory (MiPI), Gembloux Agro-Bio Tech-University of Liège, Avenue de la Faculté 2B, 5030 Gembloux, Belgium; pfickers@uliege.be (P.F.); imen_bentahar@yahoo.fr (I.B.T.)

**Keywords:** nanomaterial, metal and gold nanoparticle, cytotoxicity, biocompatibility, standardisation, safety consideration

## Abstract

Recent advances in the synthesis of metal nanoparticles (MeNPs), and more specifically gold nanoparticles (AuNPs), have led to tremendous expansion of their potential applications in different fields, ranging from healthcare research to microelectronics and food packaging. The properties of functionalised MeNPs can be fine-tuned depending on their final application, and subsequently, these properties can strongly modulate their biological effects. In this review, we will firstly focus on the impact of MeNP characteristics (particularly of gold nanoparticles, AuNPs) such as shape, size, and aggregation on their biological activities. Moreover, we will detail different in vitro and in vivo assays to be performed when cytotoxicity and biocompatibility must be assessed. Due to the complex nature of nanomaterials, conflicting studies have led to different views on their safety, and it is clear that the definition of a standard biosafety label for AuNPs is difficult. In fact, AuNPs’ biocompatibility is strongly affected by the nanoparticles’ intrinsic characteristics, biological target, and methodology employed to evaluate their toxicity. In the last part of this review, the current legislation and requirements established by regulatory authorities, defining the main guidelines and standards to characterise new nanomaterials, will also be discussed, as this aspect has not been reviewed recently. It is clear that the lack of well-established safety regulations based on reliable, robust, and universal methodologies has hampered the development of MeNP applications in the healthcare field. Henceforth, the international community must make an effort to adopt specific and standard protocols for characterisation of these products.

## 1. Introduction

Materials manufactured as nanoparticles (NPs) have demonstrated different properties as compared to their bulk counterparts due to their extremely high surface-to-volume ratio. Indeed, as the particle size decreases, the surface area increases significantly, together with the number of the available chemically reactive sites [1]. Moreover, their optical, thermal, magnetic, and electric properties may vary according to the NPs’ size. They are considered interesting materials since they can be fine-tuned, engineered, and manufactured according to their final application [2]. Among various types of nanomaterials, metal nanoparticles (MeNPs) are particularly interesting materials due to their diversified properties that are useful for catalysis [3], disease diagnosis and treatment [4], composite material manufacturing [5], and sensor technology [6]. For instance, nanoparticles show unexpected catalytic activity, which is not found with coarse-grained materials, and thus they may be useful in green chemistry to reduce hazardous substances [7]. Recent progress in the development of nanobiosensors for medical diagnosis and treatment of diseases is based on localised surface plasmon resonance (LSPR) spectroscopy [8]. The LSPR effect is characterised by a sharp increase in the optical absorption and scattering of the electromagnetic radiation by MeNPs at a certain wavelength of the incident light. Preferably, the LSPR signal of the NPs should be located in the spectral range in which the electromagnetic wave has maximal ability to penetrate the irradiated tissue (known as the “biological optical window”) for theranostic applications. The modification of the plasmonic absorption properties can be obtained by control of the spatial parameters (i.e., size, shape, and structure) of the NPs [9]. Indeed, plasmonic MeNPs have been explored for treating cancer by photothermal ablation therapy. This approach is based on the nanoparticles’ heat generation from light for destroying cancer cells, and requires high optical absorption and strong photothermal conversion efficiency [8,9]. Gold nanoparticles (AuNPs) are by far the most studied nanomaterials (NMs) regarding their biomedical application. Among various metal nanoparticles, AuNPs are believed to be the least toxic, and thus the most appropriate for biological/medical applications. However, due to their low rate of clearance from circulation streams and tissues, they may lead to health problems [10]. Therefore, focusing on AuNPs regarding their cytotoxicity vs. biocompatibility is of importance. Although they are chemically inert per se, they can be functionalised with biologically active organic molecules. They can directly conjugate and interact with proteins, nucleic acids, drugs or dyes, antibodies, and enzymes, and thus they are endowed with specific properties [11]. The array of AuNP applications is huge, ranging from clinical, pharmaceutical, and biomedical research to their utilisation in microelectronics and food packaging [12]. Herein, we will detail how their properties can modulate the effect on biological materials, more precisely in terms of biocompatibility and cytotoxicity. The most relevant in vitro and in vivo assays that might be implemented for MeNP bioapplication development will also be presented. Finally, the main regulations and requirements, as well as the main guidelines and standard methodologies to characterise new nanomaterials as nanomedicine, will be discussed. 

## 2. Cytotoxicity and Biocompatibility: Definition and Criteria

**Figure 1:**
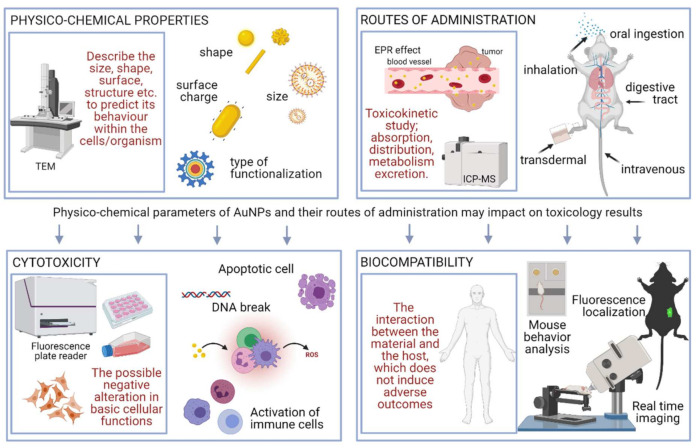
Schematic presentation of the various aspects of AuNPs (i.e., physicochemical characteristics and routes of administration) that impact their cytotoxicity and biocompatibility. For instance, size, shape, and the path by which the AuNPs are taken into the body (oral, transdermal, etc.) strongly influence toxicology and may cause adverse effects (apoptosis, ROS production, abnormalities in behaviour, etc.). These aspects may be measured on the cell or organism level. EPR, Enhanced Permeability and Retention effect; TEM, transmission electron microscope; ICP-MS, inductively coupled plasma mass spectrometry. Figure was created using Biorender (https://biorender.com, accessed on 6 October 2021).

While AuNPs have emerged as promising tools for a wide range of biomedical applications, their extensive use depends on the assessment of their biosafety. There is a growing demand to evaluate the health impact of these materials and to expand knowledge of their toxicity and biocompatibility [13]. When a novel nanomaterial emerges, its cytotoxic effect; i.e., the possible alteration of basic cellular functions, is usually evaluated at first. However, a lack of cytotoxicity does not confer these materials an implicit biocompatibility. This must be assessed as a separate criterion. The biocompatibility concept is based on the adequate interaction between the material and its biological environment; i.e., the absence of toxic or immune response from the treated biomaterial (cell, tissue, or organism) [14,15]. Very frequently, biocompatibility has been described as the property of a specific material/device to be compatible with a living tissue or organism (Figure 1). In general, a biocompatibility is obtained when the interaction between the nanomaterial and the host does not induce adverse outcomes such as oxidative stress, DNA damage, mutagenesis, or apoptosis [16,17]. Cytotoxicity is usually related to the negative impact on a specific cell line (Figure 1). Thus, cytotoxicity is usually first evaluated by specific assays performed in vitro prior to in vivo tests. Whether in vitro or in vivo methods are used, results of investigations conducted regarding AuNPs’ toxicity are currently contradictory.

In fact, it has been observed that cytotoxicity and biocompatibility are governed by several factors, which include the inherent physicochemical properties of the AuNPs and how they are delivered into the body (Figure 1). For instance, a higher toxicity of the AuNPs was found when oral and intraperitoneal administration was performed as compared to an intravenous injection [18]. Moreover, the biocompatibility was highly tissue or organ specific [14]. Nanoparticle cytotoxicity is also strongly related to physicochemical characteristics such as size, shape, surface charge, and aggregation state [19,20,21]. Taken together, all these highlight that nanoparticle biocompatibility depends closely on several factors, which span from the intrinsic properties of particles, formulation, biological target, dose, and even the methodology employed to evaluate their toxicity. Hence, the determination of an absolute nanoparticle’s biocompatibility label based on standard protocol is misleading. It must be evaluated independently in a cell/tissue and a specific application manner [22].

### Impact of NPs’ Size/Shape/Functionalisation on Their Biological Activity

The size of AuNPs considerably modulates their uptake by cells and their cytotoxicity. AuNPs penetrate cells mainly via clathrin-mediated endocytosis, and exit cells via exocytosis [13,23]. The trend for increased uptake of smaller particles may be explained by assuming that smaller particles require less energy to be internalised and must interact with a lower number of cell surface receptors compared with larger particles, and thus, this may impact their toxicity. Conversely, for larger particles, there may be insufficient receptors on the cell surface, which may limit their internalisation [13]. Generally, the smaller the size of nanoparticles, the higher the cytotoxic effect [24,25]. A size-dependent cytotoxicity study of polyethyleneglycol (PEG)-coated AuNPs in mice has been reported [26]. Particles with sizes ranging between 10 and 60 nm exhibited an adverse effect, such as alteration in cell shape, inhibition of their proliferation, or mutation in DNA, while those with sizes ranging between 5 and 30 nm had no toxicity. In fact, in mice treated with 10–60 nm particles, alanine transaminase and aspartate transaminase levels increased significantly, indicating liver and kidney damage by AuNPs. In addition to this, Pan et al. reported that exposure of L929 fibroblasts or cervix carcinoma epithelial cells (HeLa) to 1.4 and 15 nm AuNPs yielded necrosis for the smaller AuNPs, while the larger ones were shown to be noncytotoxic [27]. Although numerous studies have suggested that smaller nanoparticles are more toxic, others reported a higher cytotoxic effect for larger AuNPs [28,29]. Kim et al. even reported that AuNPs’ size did not significantly affect their toxicity Hence, no simple conclusion could be drawn from the data reported in the literature, and therefore it is difficult to establish a systematic relationship between nanoparticle size and toxicity [30]. One of the hypotheses that could explain the toxicity of small-sized AuNPs is that it is a result of the presence of a high surface area relative to their volume. This leads to enlarged absorption capacity, and may increase the chance of interacting with biomolecules [31].

Nanoparticle shape is a structural feature that can also modulate their cytotoxicity [13,32]. AuNPs with various shapes (spherical, rods, triangle, star, octahedron, plate, and prisms shapes) have been synthesised and assessed for their cytotoxicity. Steckiewicz et al. investigated the cytotoxicity of rods, stars, and spherical AuNPs on human fetal osteoblast hFOB 1.19 and pancreatic duct cell hTERT-HPNE cell lines [24]. AuNP rods were the most toxic to human cells, while AuNP spheres appeared to be the safest. Wang et al. reported that AuNPs nanorods were highly toxic in comparison to AuNP hexapods [33]. This highlighted that the cytotoxicity of AuNPs was shape-dependent. According to the different shape, the distribution of the surface atoms in AuNPs may have changed. Thus, some explanation may be offered by considering the geometry of a sphere in relation to the stars or rods. More atoms at angles and edges may cause stronger interactions with biomolecules. Therefore, the rods and stars (featuring a larger amount of edges and corners) showed toxicity, which is often not observed for spherical nanoparticles [7,31].

AuNPs can be functionalised with different molecules in order to prevent their aggregation in biological fluids, facilitate their cellular uptake, or improve their specificity [34]. A variety of ligands such as peptides, antibodies, PEG, citric acid, cetrimonium bromide (CTAB), nucleic acids, and drugs have been used to design AuNPs with appropriate and specific characteristics. Moreover, the AuNPs’ cytotoxicity could be fine-tuned by using these ligands. Bhamidipati et al. stated that the surface chemistry of coated AuNPs had a predominant effect on their cytotoxicity [35]. CTAB-capped AuNPs were found to be the most toxic to human dermal fibroblasts in comparison to PEG and serum-capped AuNPs. According to Arvizo et al. the surface ligands played a key role in shaping and defining the characteristic of the coated AuNPs, which in turn affected their interaction with cells [36]. A related concept of “cell vision” has been suggested to explain the effect of ligands on nanoparticle uptake and cytotoxicity. This concept was firstly introduced for magnetic nanoparticles before being expanded later to include other types of nanoparticles. Cell cytoplasmic membranes are characterised by their specific surface molecules, which are composed of various proteins, sugars, and phospholipids. The interaction between nanoparticles and these specific structures, which are dependent on cell type, traduces how a given cell type sees these nanoparticles. Therefore, the specific interaction between nanoparticles and cell surface moieties yield to different cell responses, and considerably affect the cytotoxicity of AuNPs [37,38].

Surface charge is another parameter that considerably affects the behaviour of nanoparticles, such as their interaction with cell membranes or proteins and their stability in biological fluids [39,40]. Goodman et al. reported that at the same concentration, cationic AuNPs were moderately toxic, while anionic AuNPs were nontoxic for erythrocytes [41]. The same authors investigated the effect of AuNPs with different surface charges on zebrafish embryo development [41]. Noncharged AuNPs did not induce adverse effects, whereas negatively charged AuNPs provoked abnormalities in larval behaviour. Generally, cationic surface charge is responsible for binding plasma proteins and forming the so-called “protein corona”, which is then taken up by macrophages. Thus, cationic AuNPs are much more potent in generating immune response than anionic or neutral NPs [42,43]. It should be pointed out that interaction of AuNPs with cells should not be expected as basic electrostatic interactions only, but other short- and long-order forces, such as hydrophobic interactions, hydrogen bonding, and ligand−receptor interactions, play key roles in the cellular uptake [13].

The aforementioned properties, as well as aggregation state, dose, and interaction with physiological media, may affect AuNPs’ cytotoxicity. Yang et al. reported that aggregated cationic AuNPs were fourfold more toxic to human dermal fibroblasts cells in comparison to nonaggregated particles. A number of coating agents such as lipids, polymers and proteins form a protective layer on AuNPs, thus preventing their aggregation [44,45,46]. Additionally, the dose of administered AuNPs to a model organism may also affect their toxicity. Recently, Coradeghini et al. revealed that AuNPs’ toxicity against mouse fibroblasts cell lines was dose-dependent [47]. After their uptake, AuNPs interact with various biological components that influence the physicochemical characteristics of AuNPs, such as aggregation state and surface charge [48,49]. Additionally, some proteins from the biological media, such as bovine serum albumine (BSA), cytochrome c, fibrinogen, and ubiquitin, could be easily adsorbed onto the surface of AuNPs and form a protein corona. This interaction could trigger different physiological and pathological changes such as apoptosis, the increase of lysosomal permeability, and inflammatory responses [49,50].

It is also worth noting that the physicochemical properties of NPs strongly influence the uptake and intracellular trafficking of NPs. For AuNPs to be wrapped or penetrate through the cell membrane, the ligand–receptor, surface charge, hydrophobicity, size, and shape-binding interactions must overcome the resistive forces associated with membrane stretching and elasticity. Generally, AuNPs can cross the cell membrane through two possible routes: endocytosis and direct penetration. Although clathrin- and caveolin-mediated endocytosis are the main uptake mechanisms for AuNPs, others such as flotillin-mediated endocytosis, phagocytosis, and pinocytosis were also reported [51]. The uptake mechanism for one and the same NP into different cell types may even vary [52,53]. In addition, in the same tested cell line, diverse internalisation mechanism may occur. For instance, two uptake mechanisms of gold and iron oxide NPs, caviculae- or clathrin-dependent systems, respectively, have been described [54]. The caveolae are characteristic flask-shaped membrane invaginations, lined with caveolin protein, and enriched with cholesterol and sphingolipids. The clathrin-mediated endocytosis typically occurs in a membrane region enriched in clathrin, which is a main cytosolic coat protein [51]. 

Due to increasing applications of AuNPs in nanomedicine (for review, see [11]), their nanotoxicity must be initially assessed. This can be performed using in vitro and in vivo assays, which are described in the next section. 

## 3. Methods for Toxicity Evaluation

Both in vitro and in vivo toxicity evaluation of AuNPs have advantages and disadvantages, but both are necessary to understand the impact of nanoparticles on living organisms. In vitro methods allow faster, simpler, and cost-efficient assessment of the toxicity. However, they do not consider the complexity of multitissue organisms such as mammals. In contrast, in vivo evaluation gives a more detailed view of AuNPs’ systemic toxicity. However, they are time-consuming, expensive, and may present ethical issues. As an alternative, data from in vitro tests are used to extrapolate what might be expected at a systemic level (organs or organism). The following sections point out and briefly describe some of those procedures, either for in vitro or in vivo models, used to assess the toxicity of AuNPs. Nevertheless, this list does not imply that each of the methods are necessary, nor does it indicate that only these methods are currently available.

### 3.1. In Vitro Methodology

Although in vitro assays are not able to provide a complete perception of the biocompatibility of AuNPs, they have a paramount importance, as they provide reliable basic data on the safety of these NPs [55]. The most commonly used in vitro assays aim to explore cell proliferation to measure some metabolic functions, or to assess the effect on the genetic material (summarised in Table 1). A wide variety of cell types, both microbial (bacteria and fungi) and higher eukaryotes, can be used as a model to conduct in vitro toxicity assessment. For higher eukaryotes, both primary cells (i.e., cells taken directly from animal models) or cell lines (i.e., immortalised cells) can be considered [56]. Moreover, the high proliferation rate of cell lines makes the in vitro approach very popular for a first toxicity screening [31,47,57,58]. There are a number of cell lines that have been used to assess the effects of AuNPs [31]. In general, the key to choosing the model cells is to consider the purpose of the research, which depends on the problem it seeks to address. For instance, while studying the particular disease; i.e., cancer, the model of cells that closely exemplifies this disease must be chosen [59]. A good resource to find a proper cell line suitable for the experiment is the Cancer Cell Line Encyclopedia (CCLE), which provides public access to genomic data and analysis for about 1000 cell lines [60]. However, the outcomes from different in vitro toxicological studies are scattered, mainly because they were performed most of the time in different experimental conditions; i.e., various types of cells.

Metabolic activity and cell proliferation rate or cell viability are the most commonly used parameters for a first estimation of the toxic effect of a nanomaterial. Diverse assays (colorimetric, fluorimetric, or luminometric), based on the various cell functions; i.e., enzyme activity, cell membrane permeability, adherence, or ATP production, are performed to assess metabolic cell activity [71]. The principle of those assays is the measurement of biochemical markers to evaluate metabolic activity of the cells or the number of cells. The 3-(4,5-dimethylthizol-2-yl)-2,5-diphenyltetrazolium bromide (MTT) assay is the gold standard for determination of cell viability and proliferation. It is based on the reduction of MTT into formazan crystals by dehydrogenases in mitochondria or endoplasmic reticulum of living cells. While MTT gives a water-insoluble formazan, which must be dissolved before colorimetric quantification, other tetrazolium-based assays, such as XTT (2,3-bis(2-methoxy-4-nitro-5-sulphophenyl)-5-carboxanilide-2H-tetrazolium, monosodium salt), MTS (5-(3-carboxymethoxyphenyl)-2-(4,5-dimethyl-thiazoly)-3-(4-sulfophenyl) tetrazolium salt), and WST (2-(4-iodophenyl)-3-(4-nitrophenyl)-5-(2,4-disulfophenyl)-2H tetrazolium monosodium salt), allow a direct quantification [71]. However, it must be highlighted that tetrazolium reduction reflects the general cell metabolism rather than the cell number. The rate of MTT reduction can change with cell physiology or culture conditions [72]. An alternative dye to tetrazolium salts, alamar blue, which is a nontoxic fluorogenic dye, can be used for long-term cell proliferation study [73].

AuNPs are known to be able to disrupt cell membrane integrity. Thus, monitoring the leakage of specific cell components such as lactate dehydrogenase assay (LDH) or adenosine tri-phosphate assay (ATP) in specific assays can be used to assess AuNPs’ cytotoxicity. When cells are damaged, there is a leakage of lactate dehydrogenase, which can be further quantitatively measured and compared to the control cells [74]. Cell adhesion (the ability of cells to fix a solid support) can be impaired by AuNPs, and thus can be used as a parameter to estimate their cytotoxicity. Estimation between the living/adherent and dead/detached cells can be performed using simple crystal violet staining. This dye can easily bind to the proteins and DNA only of viable cells [71]. It worth mentioning that in such assays, the optical properties of AuNPs and absorption ability may affect the test quantification [75].

Oxidative stress resulting from exposure to AuNPs leads to the production of reactive oxygen (ROS) and nitrogen species (RNS). The key factors involved in NP-induced ROS include: (i) pro-oxidant functional groups on the surface of NPs; (ii) active redox cycling on the surface of NPs due to transition metal-based NPs; and (iii) particle–cell interactions [76]. Through this, AuNPs can generate damaging radicals, and participate in redox reactions as an electron acceptor or donor. They may directly interact with specific molecules (i.e., DNA, proteins, and lipids), subsequently leading to cell membrane damage, protein degradation, and mutagenesis [77]. Some evidence also has suggested that AuNPs can act as both an antioxidant and pro-oxidant agent, depending upon the concentration and target cells [78]. Oxidative stress is mainly assessed by a DCFDA assay [55]. In this method, the nonfluorescent compound 2′,7′-dichlorofluorescin diacetate (DCFH-DA) enters cells. In the presence of ROS/RNS, it is easily oxidised into a highly fluorescent compound, 2′-7′-dichlorofluorescein (DCF). Measurement of this fluorescence is used to quantitate ROS/RNS [79]. However, the availability of other oxidative stress biomarkers makes the oxidative stress measurements even more accurate and convenient. For instance, malondialdehyde, a major marker of lipid peroxidation, can be quantified using a TBA (thiobarbutiric acid) assay [80]. Other lipid peroxidation markers such as 4-hydroxyl-2-nonenal, isoprostane can be quantified by an enzyme-linked immunosorbent assay (ELISA) [81]. ROS may also react with protein backbone or amino acid side chains and lead to the formation of proteins carbonyls, which can be detected by immunoblotting, ELISA, or chromatographic methods [82]. The exposure to NMs can also impair the activity of different antioxidant enzymes such as superoxide dismutase, catalase, and glutathione S-transferase. Therefore, different techniques have been discussed in literature to assess antioxidant enzymes activities [79,83]. 

Apoptosis (i.e., programmed cell death) and necrosis (i.e., accidental cell death) are two different mechanisms by which cells can die in response to excessive oxidative stress. The differentiation between those mechanisms can be easily identified by changes in cell morphology or by agarose gel electrophoresis. In the latter one, chromatin cleavage and nucleosome-sized DNA fragments are a hallmark of apoptosis. Thus, analysis of the electrophoretic profile gives a distinct DNA fragment for apoptosis, but a smear of DNA in the case of necrosis [84]. Among different apoptotic pathways, the intrinsic mitochondria-mediated pathway plays a major role in metal–oxide NP-induced cell death [76]. Mitochondria-related apoptosis is elicited by upstream ROS production, leading to mitochondrial dysfunction and subsequently inducing apoptosis. In this case, the upregulation of p53 (proapoptotic gene), downregulation of Bcl-2 (antiapoptotic gene), Bax translocation, and cytochrome c release is observed [66]. Flow cytometry is also considered as a convenient tool to evaluate and discriminate cell death induced by AuNPs [27]. Propidium iodide (PI) and annexin V are often used to determine if cells are viable, apoptotic, or necrotic through the differences in plasma membrane permeability. The live cells are not stained due to the presence of an intact plasma membrane. Early apoptotic cells, with the phosphatidylserine translocated to the outer leaf of the plasma membrane, are stained with annexin V due to its high affinity to the negatively charged phospholipid of phosphatidylserine. In the necrotic cells, the plasma and nuclear membranes decrease, and thus, PI may intercalate into the DNA and stained cells. Indeed, Pan et al. reported on AuNPs’ toxicity on different cell lines, performed with a double-staining annexin V/PI assay [27]. This method involves the fluorescence-based detection of counterstaining via laser-beam-employing instruments, including a flow cytometer; however, the other fluorescence microscope and automated cell counter methods are useful [85]. The examination of membrane integrity of model cells after exposure to MeNPs is an important parameter considered in the type of cell death. Membrane integrity also can be evaluated using a neutral red assay and trypan blue exclusion test. Trypan blue, a membrane-impermeable dye, is excluded by viable cells and taken up dead cells [71]. 

Like other MeNPs, AuNPs may also interact with immunocompetent cells and induce direct or indirect damages [86]. Direct immunotoxicity leads to apoptosis or necrosis, while indirect damages concern changes in immune cell functions measured by alteration of surface marker expression, cytokine production, cell differentiation, and immune activation. Therefore, evaluation of immunotoxicity is mandatory for the safe use of AuNPs [87]. Although not specifically defined for AuNPs, recommendations and guidelines from organisations such as the Organization for Economic Co-operation and Development (OECD), the European Food Safety Authority (EFSA), and the International Council for Harmonisation of Technical Requirements for Pharmaceuticals for Human Use (ICH) for testing chemicals are used to assess NM-induced genotoxicity [88]. The specific immune response is assessed by the measurement of lymphocyte proliferation exposed to NMs using _3_H-thymidine incorporation. Thymidine is incorporated into dividing cells, and then the level of radiation is measured. Cytokines are recognised as biomarkers to predict immunotoxicity of engineered AuNPs. Both inflammatory and immunoregulatory cytokine expression can be determined by ELISA assay, flow cytometry, and RT-qPCR [87]. The function of phagocytes, as the key component of the immune response, can be determined by the phagocytic activity of granulocytes and monocytes. The principle of this assay is to determine the phagocytic activity of cells by measuring the fluorescence. The fluorescence appears from the fluorescein-labelled *Staphylococcus aureus*, which are phagocyted by the cells, and then the signal can be measured with flow cytometry. Cytolytic function of natural killer cells can be monitored by radiolabel release or flow cytometry [87]. 

Genotoxicity testing is a crucial criterion in the safety evaluation of engineered nanoparticles, and one of the most important impacts of AuNP action seems to be the ability to cause DNA damage. It may be caused directly by binding protein or DNA, or indirectly through the generation of ROS [89]. The in vitro genotoxicity tests include the bacterial reverse mutation (Ames) assay, mammalian assays, chromosomal damage, micronucleus induction, and DNA strand break [90]. The Ames assay uses several bacterial species (i.e., *Salmonella* spp., *Escherichia coli*) that carry a specific reverse mutation in the histidine synthetase gene. Reverting of this mutation, caused by an immunotoxic agent, restores the amino acid prototrophy, enabling the bacteria to grow on histidine-free media. Although this assay is easy to conduct, it suffers from a lack of reliability due to the size of NMs (sometimes larger than bacteria) and the presence of bacterial cell walls, which limit NMs’ entrance into cells [91,92]. Mammalian cells are considered more suitable to assess AuNPs’ genotoxicity. According to standard assays, mutations are assessed at specific locus using specific cell lines, often the TK (thymidine kinase) gene in L5178Y cells (OECD TG 490), and the Hprt (hypoxanthine guanine phosphoribosyl transferase) in V79, CHO, CHL, L5178Y, and TK6 cells (OECD 476) [93]. A micronucleus is a small nucleus formed during the anaphase stage from chromosomal fragments left behind when the nucleus divided. Micronuclei are caused by DNA aberration as a response to natural processes, or many cytotoxic factors. Different types of micronuclei assays, such as cytokinesis-block micronucleus assay, mammalian erythrocyte micronucleus assay, and buccal micronucleus assay are considered for the assessment of NMs genotoxicity. The comet assay (CA), also known as single-cell gel electrophoresis (SSGE), is widely used for the assessment of AuNPs’ genotoxicity by measuring DNA damage in eukaryotic and some prokaryotic cells [94]. The technique uses cells that are suspended in a thin agarose gel on a microscope slide. After the cells have been lysed, the DNA is denatured, electrophoresed, and stained with DNA-binding dye (i.e., silver stain, ethidium bromide, propidium iodide, YOYO-1, or SYBR gold). Cells with DNA damage show increased migration of chromosomal DNA from the nucleus towards the anode, resulting in images similar to comets [95,96]. Epigenetic modification is a novel toxicity pathway considered to identify links between MeNP exposure and epigenetic modifications. Emerging studies have noticed that nongenotoxic agents can induce epigenetic alterations, including the changing of DNA methylation, modification of histone, and miRNA expression. Epigenetic toxicology can be assessed by combining standard molecular biology techniques and whole-genome approaches [97,98,99]. Aberrant DNA methylation is the most studied criteria in epigenetic testing. A set of different methods, such as methylation-specific PCR, medium throughput comet assay, chromatin precipitation, whole-genome bisulfite treatment with sequencing, combined bisulfite restriction analysis for gene-specific DNA methylation, whole genome bisulfite treatment with sequencing, and methylated DNA immunoprecipitation, are considered to analyze DNA methylation [98,99]. Moreover, microarrays, real-time PCR, and in situ hybridisation are used to analyze microRNA [100,101].

### 3.2. In Vivo Methodology

A preliminary estimation of NPs’ toxicity can be deduced from in vitro tests. However, realistically, it is not congruent to match the laboratory results to complex biological interplay, which is found in in vivo models. Therefore, assessment of NMs must involve a thorough biocompatibility testing program, which typically comprises the effects on organs and the immune system [102]. Any nanomaterial that may be implemented in the human body must be investigated to determine whether it penetrates cells/tissues, and how it is distributed, biodegraded, and excreted. Thus, using animal models is unarguably important, and should not be excluded from preclinical toxicity studies of nanoformulations [103]. On the other hand, keeping in mind the 3Rs (refine, reduce, and replace animal testing), the testing strategy should reduce the number of animals [104].

A systemic toxicity assessment of AuNPs is usually performed on animal models such as mice or rats when bioapplication is considered [55]. The major routes for introducing the particles into the body are intravenous, subcutaneous, and oral. Afterward, they can be distributed to various organs, where they interact with biological components and can be modified or metabolised. To conclude, many factors impact the nanotoxic effect of AuNPs, and thus, they must be thoroughly examined in the face of application into the body [105]. Among different in vivo assays (summarised in Table 2), the toxicokinetic study, an all-encompassing process consisting of absorption, distribution, metabolism, and excretion (ADME), is essential for understanding the toxicity of an NM.

To assess the toxic effects of AuNPs on animals, their behaviour and body-weight changes can be monitored. Normal activity without lethargy or apathy after NP administration, as well as no significant changes in their food consumption and weight, suggest no negative impact of the nanomaterial [107]. However, more specific methods should be further performed. Among these assays, biodistribution examines the localisation route of AuNPs to the tissues and organs. After inoculation of the AuNPs into the model organism, the biodistribution of the material is assessed over time. The tissues/organs and subcellular localisation can be monitored directly, through the electron or fluorescence microscopy imaging, or indirectly with assessment of the content of Au using the inductively coupled plasma mass spectrometry (ICP-MS) technique) [107]. However the latter method has a limitation, as the presence of the chemical element (gold) is determined, not the presence of nanoparticles themselves [108]. AuNPs can be fluorescent-labelled to characterise their behaviour in real-time and to evaluate their biodistribution. For instance, citrate-stabilised gold nanoparticles were PEGylated and then tagged with fluorophores. Covalent attachment of the fluorophore was also confirmed with agarose gel electrophoresis. The results showed that appropriately engineered fluorescent-tagged gold nanoparticles were enabled in multicolor in vivo imaging, and thus their biodistribution could be visualised [109].

Other factors that must be considered when AuNPs are used in humans are the pharmacokinetics and clearance of the nanomaterials. This is important, especially for AuNPs’ targeted-delivery system, in which nanoparticles act as a carrier for delivering a drug. NPs have been found to accumulate in tumor tissues through a passive mechanism, known as the enhanced permeability and retention effect (EPR) [110]. A large part of a solid tumor possesses a leaky vasculature, with enhanced permeability to increase the supply of nutrients and oxygen necessary for their rapid growth. AuNPs can pass through the fenestration in the blood vessel endothelium and accumulate in the tumor site [111,112]. To determine the pharmacokinetic profile and the half-life of the AuNPs, samples of the blood are usually collected during the testing period of the experiment, and the content of gold is quantitatively estimated. Clearance of the nanomaterials may be estimated similarly to the clearance of the drug, which is defined as the volume of blood cleared of drug per unit time [113]. The liver and the kidneys are the two main routes for nanomaterial clearance, and the type of route depends on the size of the NPs [114]. When AuNPs are larger than renal filtration cutoff, they are excreted from the blood by the reticuloendothelial system, and they accumulate in the liver. In general, elimination routes include: (i) renal excretion (urine), which involves the filtration of small particles within hours to days after administration; (ii) hepatobiliary excretion (bile to feces) within a few hours to weeks after administration; and (iii) the reticuloendothelial system, which involves the trapping of nondegradable nanoparticles in the body for a prolonged period (>6 months) [31]. As a consequence, the kidney, liver, and spleen are the main organs for the investigations of AuNPs’ metabolism and clearance. Generally, localisation of the AuNPs in organs is performed through optical imaging. However, due to the limitation of tissue depth penetration and low resolution, providing other methodologies or devices is still desired. For example, single-photon emission computed tomography (SPECT) or dynamic positron emission tomography (PET) are the techniques that are capable of deep tissue penetration and visualizing the dynamic process of AuNP distribution in vivo, respectively [115]. Penetration and accumulation of gold nanoparticles can be also monitored using a surface-enhanced Raman scattering (SERS) technique. Therefore, SERS spectroscopy has the potential to monitor the dynamics of nanoparticle penetration and accumulation within the tissues [116].

After administration, AuNPs interact directly with blood components. They can alter haematologic factors, and induce an inflammatory or immune response. Due to this, the first biochemical and haematology factors that must be determined are the red and white blood cells, haemoglobin, haematocrits, and platelets. Haematology results depend strongly on the concentration of administered AuNPs and the routes of their distribution. It has been noticed that the tail vein injection presents the lowest toxicity, while the oral administration route induces the highest toxicity, with damage to the gastrointestinal system, which has further effects on the immune system via splenic metabolism [18]. In the case of impact of the AuNPs on the inflammatory response, the standard biochemical parameters such as alanine aminotransferase (ALT), aspartate aminotransferase (AST), total bilirubin (TBIL), albumin (ALB), and gamma-glutamyl transpeptidase (GGT), are determined in the blood serum collected during the experiment. Usually, these assays are performed with an enzymatic colorimetric test [117]. Furthermore, the determination of the proinflammatory parameters, such as cytokins or chemokines, as well as the level of immunoglobulins, could be measured using a specific antibody assay; i.e., ELISA [21,118,119]. Another factor that must be considered during the evaluation of AuNPs’ biocompatibility is their immunotoxicity, including immunostimulatory and immunosupressive effects (review in [120]). Immunotoxicity assays are commonly performed through immunohistochemistry methods that consist of the detection of a specific antigen–antibody interaction in paraffinised sections of the tissue; i.e., expression of proinflammatory cytokines [121,122]. Beside this, RT-qPCR can be used as a more sensitive assay to evaluate the immunocompatibility by quantification of the mRNA expression level of the interleukines (IL-1β, IL-6) [123]. 

Histopathological examination of organs allows the assessment of the potential AuNP-induced tissue damage. Fixed specimens of the tissue taken from animals exposed to AuNPs are cut, stained, and observed under a microscope. Histological changes in major organs (e.g., liver, kidney, or spleen) are preferably observed by hematoxyline/eosine staining, and are recognised as steatosis, vesicles, cytoplasmic degeneration, necrotic foci, haemorrhage, infiltration of inflammatory, presence of granular leukocytes and giant macrophages, diminished and distorted glomeruli, and dilated tubules [124]. 

Similar to other drugs or pollutants, MeNPs may have negative effects on reproductive organ function and development. The potential adverse effect of AuNPs on the reproductive system has been investigated by histological observations and assessment of AuNP accumulation in male or female reproductive organs, evaluation of the sperm quality and fertility of males, as well as the quantification of the sex hormones in the serum of the blood. More specifically, AuNPs can accumulate in the testes and cause the reduction of testosterone production through the downregulation of the expression of 17α-hydroxylase [125,126]. 

The European Medicine Agency (EMA) issued a guidance, and recommended standard genetic toxicology assays, both in vitro and in vivo, to determine the carcinogenic effects of pharmaceuticals [127]. Based on this recommendation, genotoxicity and mutagenicity may be assessed by the micronucleus test and comet assay, which are suitable to evaluate DNA damage [94,127]. Although these methods are not yet validated, authorities such as the EFSA promote them. They suggest including them in test strategies to assess genotoxicity in vivo in an organ other than bone marrow or liver. The CA test is commonly used in the in vitro approach (described above); however, it also can be performed in vivo by cell dissociation from the tissue [108,128].

One of the aims of performing in vivo testing is to determine the dose–response relationship, and either an acute or repeated-dose study can be introduced here. Prior to any potential therapeutic applications of AuNPs, studies to evaluate acute or subacute, as well as chronic or subchronic toxicity, must be conducted. Oral toxicity may be assessed by repeated oral administration of the material/drug during a limited period (one dose level daily for 28 or 90 days). Based on these guidelines, evaluation of the health hazards after exposure to AuNPs may be also investigated in inhalation toxicity studies [129].

## 4. Safety Considerations

Although the current trends of using nanoparticles in biomedical applications appear promising, methods and models to measure pharmacokinetics, biodistribution, and toxicity require more attention, especially when administered to humans. For this purpose, designed nanomaterials should be either small enough to pass through biological barriers and enter the cells, or large enough to load appropriate amounts of specific components on them [130,131]. Due to the complex nature of nanomaterials, conflicting studies have led to different views of their safety. As described above regarding some useful applications of AuNPs, the general requirements are biocompatibility and biological safety. So far, reviews of nanomaterials’ toxicity have been published in many journals, and most of them are a list of reports from research. Usually, they have focused on NP–cell/organism interactions in a specific and restricted environment. Therefore, the in vitro studies cannot be directly compared to in vivo studies, as reported in many articles. In vitro experiments are usually conducted with a high dose of NPs, and direct extrapolation of those results to in vivo studies is not viable, since nanoparticles will gradually be cleared by renal and fecal excretion [132]. Therefore, for the evaluation of nanotoxicity, the doses used in vitro should be low [133]. Moreover, defining the NP dose as a concentration is not suitable to determine the dose–response relationship, as it is for conventional chemicals or drugs. Other metrics; i.e., number of NPs or surface area, are likely to be more appropriate [134]. Another drawback of properly assessing the toxicity of NMs explored by various researchers and laboratories is using different and often not comparable methods. Thus, the characterisation of nanomaterials must be comprehensive and broad in scope. Moreover, an interdisciplinary approach to the assessment of the toxicity is required. Therefore, in the following sections, we will point out some regulations and methods according to international standards that should be taken into consideration when nanotoxicity/nanosafety of new materials is assessed.

## 5. Regulation

The rapid advances in nanotechnology have resulted in a flood of different nanoparticles developed for various biomedical applications. Since the European Commission (EC) elaborated the first definition of nanomaterials, great efforts have been made to find a specific approach to assess the nanorisk associated with their industrial implementation [130,135]. In this section, we will focus on the present legislation and requirements given by regulatory authorities to investigate fundamental characteristics of nanomaterials.

### 5.1. Regulating Institutions

Currently, the European requirements for all substances (used as pharmaceuticals, food, medical devices, and cosmetics) are defined by various authorities; these are summarised in Table 3. In the U.S., the Food and Drug Administration (FDA) is responsible for establishing these regulations. Several Asian countries; i.e., China, Japan, and South Korea, have released their own standards for nanomaterial characterisation. Among South American countries, the regulations issued by ANVISA (see Table 3) play an important role in the manufacturing and regulatory landscape of nanotechnology. Summarizing the efforts in regulating the characterisation of nanomaterials, standards, and methods for their evaluation must be applied according to the type of NMs and their purposes [135]. For example, in most Asian countries, as well as in Brazil, many types of cosmetics that contain NMs are not under specific regulations. Similarities also occurred when applying nanotechnology to food products [135]. Compared to this, the European and US markets are under guidelines on how to assess the nanosafety of engineered NMs in food [108,136]. Nanomaterials targeting specific human tissues are usually classified as medicinal products, and are approved as pharmaceuticals by the EMA or the FDA (review in [135]). Thus, these NMs require specific premarket authorisation based on data provided by manufacturers, which include the physicochemical characterisation, toxicological, and ecotoxicological properties [137]. In 2000, the National Institutes of Health (NIH) began the National Nanotechnology Initiative (NNI) (creating a new definition of “nanopharmaceuticals”) as a program to promote nanoscience-related research and development [138]. Currently, the key challenges for researchers, industry, and regulators are how to classify new materials and which testing protocols should be required before products become available [139].

### 5.2. Standards and Certificates

Bioapplication of nanomaterials brings humans into direct contact with MeNPs. Henceforth, it is essential for public confidence and the nanotechnology industry to assess the health risk posed by engineered AuNPs. Therefore, new and specific standardisation and certification assays (which include the evaluation of the physicochemical characteristics, sterility, pyrogenicity, biodistribution and ADME, pharmacokinetic, and in vivo and in vitro toxicity) for preclinical study of nanosafety and toxicity risk must be developed [102]. Efforts made to standardise the procedures for AuNP risk assessment are underway, and still need improvement. Currently, nanomaterials are considered in the same way as conventional chemicals. The major nanotechnology standardisation efforts are developed by the Standards Developing Organizations (SDOs) (summarised in Table 4), although none of the standards has achieved dominance yet [156].

In general, nanodrug or nanopharmaceutic approval processes are time-consuming and expensive. The entire process is estimated to span 10–15 years, with a cost for development of approximately USD 1 billion per new formula [139]. It can be separated into three major phases: the preclinical phase, which includes the discovery and characterisation; followed by the clinical phase with human trials; then the postmarketing phase [158]. Usually, the clinical step lasts the longest, and is broken down into phase I (dosing, toxicity, excretion in healthy subjects), phase II (safety, efficacy in a larger group of patients), and phase III (multicenter, randomised, placebo-controlled) trials [139]. On the other hand, the preclinical phase is the most diverse phase of drug development, especially when metal nanoparticles with their specific properties and high degree of tailorability are considered. Therefore, NPs envisioned for human use must have robust methodologies for synthesis, characterisation, quality control, and potential scale-up [159]. 

## 6. Perspective, Recent Advances, and Conclusions

Nowadays, there are enormous expectations surrounding the application of nanotechnologies in healthcare and daily life, which in turn influences the industry. An increased concern in both Europe and the U.S. is the question of the harmonisation of methodologies essential to characterising the quality requirements. The lack of unity in protocols and methodologies to characterise NMs has hampered the development of so-called nanomedicine, as well as the industry in general. Therefore, a critical role must be played by regulatory authorities to obtain more cooperative work between regional regulatory bodies [160]. 

There is still a long way to go before obtaining universal regulatory guidelines for the characterisation, evaluation, and process control of NMs. Meanwhile, as many types of assays as possible should be always implemented before clinical trials. Even though gold nanoparticles, as a core of nanopharmaceuticals, have been proven as nontoxic, it should be never forgotten to discern either the toxicity of the stabilizing compounds or capping ligands. Similarly, formulating and nanopackaging technology may cause immunogenicity. Thus, only firmly established and well-documented physicochemical characterisation and correlation between in vitro assays and their in vivo counterparts should be considered for further industrial development of NMs.

Although a significant number of nanodrugs and nanopharmaceuticals have been approved in recent decades for a variety of indications, FDA-approved materials are heavily weighted towards polymeric and liposomal NMs. At present, there is a trend towards development of inorganic and metal nanoparticles, and indeed, AuNPS are well represented in this field. However, only a few examples of AuNPs currently are under investigation in clinical trials [161,162]. According to the literature, Aurimune CYT-6091 (Cytimmune) is the first tumor-targeted nanomedicine consisting of tumor necrosis factor-α (TNF) covalently linked to pegylated colloidal gold nanoparticles [163,164]. The conclusion after finishing the phase I clinical trial was that CYT-6091 targeted tumors in humans and was well tolerated at doses greater than maximum tolerated dose for native TNF. However, further clinical studies with chemotherapy combinations are planned. Among intravenous nanoparticle therapies that are not clinically approved and are currently undergoing clinical trials are AuroLase (Nanospectra Biosciences, ClinicalTrials.gov Identifier NCT01679470) for thermal ablation of solid and metastatic lung tumors, and NU-0129 (Northwestern, ClinicalTrials.gov Identifier NCT03020017) for patients with recurrent glioblastoma multiforme or gliosarcoma [161].

Taken together with all these recent approvals and trials, the field of AuNPs used as nanomedicine continues to make breakthroughs that improve human health. However, due to their unique physicochemical and optical properties and their promising potential in bioapplications, the international community must make an effort to perform specific protocols for preclinical development and characterisation of these products.

## Figures and Tables

**Table 1:** In vitro assays and their principles subdivided into a group that measures specific cell response.

Type of Study	Principle of the Method and Application
Metabolic activity	Used to measure cellular metabolism by assessment of metabolically active cells [61].
Cell proliferation and viability	Measures the balance between cell divisions and cell death in a response to NMs stimuli or assessment of the ratio of the live to dead cells [62].
Oxidative stress	Measures the imbalance in free radical formation within a cell caused after exposure to NMs [63].
Apoptosis assays	Analyses whether the cells can trigger their own death in response to extracellular signals such as MNs [64].
Necrosis assays	Used to evaluate membrane integrity and determine the viability of cells [65,66].
Genotoxicity	Identification of the damages of genetic information within a cell causing mutations after NM treatment [67,68].
Immunotoxicity	Measures the immunomodulatory potential of the NMs; may be exhibited as either suppression or enhancement of the immune response [69,70].

**Table 2:** Types of in vivo studies and their main principles used to assess the biocompatibility of NMs [106].

Type of Study	Principle of the Method and Application
Behavioural analysis and body weight	Investigation of changes in animal behaviour and body weight after exposure to AuNPs.
Biodistribution	Shows the localisation of AuNPs in tissues and organs; can be detected in live or killed animals.
Biodegradation and clearance	The examination of excretion and metabolism of nanoparticles at various time points after exposure.
Pharmacokinetic, haematology, and serum chemistry	Analysis of the components of the blood; estimation of the blood half-life of NMs.
Immunology	Evaluates the potential side effects of NMs, and includes inhibition or enhancement of the immune response; histopathology of lymphoid organs.
Histopathology	Examination of tissues exposed to NMs, with their localisation and identification of pathological changes in the structure of tissues.
Acute and repeated-dose toxicity	Describes the adverse effects of a substance that result either from a single exposure or from multiple exposures in a short period of time.
Reproductive and developmental toxicity	Defined as adverse effects of a chemical substance on sexual function and fertility in adult males and females, as well as developmental toxicity in offspring.
Genotoxicity and mutagenicity	Impact of NMs on genetic materials and evaluation of the induction of permanent transmissible changes in the amount or structure of the genetic material of cells or organisms.

**Table 3:** Regulating authorities that have defined specific requirements for various groups of (nano)products.

Name of the Authority	Regulations, Procedures, Standardisation, and References
Joint Research Centre (JRC)	Aims to provide evidence-based scientific support in the European policymaking process [140].
European Chemicals Agency (ECHA)	Manages the implementation of all the regulations on registration, evaluation, authorisation, and restriction of chemicals (REACH) [141].
Scientific Committee on Emerging and Newly Identified Health Risks (SCENIHR)	Provides opinions on emerging or newly identified health and environmental risks; and on broad, complex, or multidisciplinary issues requiring a comprehensive assessment of risks to consumer safety or public health and related issues [142,143].
Scientific Committee on Consumer Safety (SCCS)	Provides opinions on health and safety risks (chemical, biological, mechanical, and other physical risks) of nonfood consumer products (e.g., cosmetic products and their ingredients, toys, textiles, clothing, and personal care and household products) and services [144,145].
European Food Safety Authority (EFSA)	Publishes guidelines on the evaluation of nanosafety in food products, with recommendations for analytical technologies [146,147].
European Medicines Agency (EMA)	Fosters scientific excellence in the evaluation and supervision of medicines, for the benefit of public and animal health in the European Union (EU) [148,149].
U.S. Food and Drug Administration (FDA)	Responsible for protecting the public health by ensuring the safety, efficacy, and security of drugs, biological products, medical devices, food supply, and cosmetics [150,151].
Quality Supervision, Inspection, and Quarantine (AQSIQ)	Responsible for commodity inspection, food safety, certification, accreditation, and standardisation [152].
Chinese National Nanotechnology Standardization Technical Committee (NSTC)	Reviews the standards for nanomaterials [153].
Standardization Administration of the People’s Republic of China (SAC)	Setting up standards for nanomaterials and nanomaterial characterisation [154].
Brazilian Health Surveillance Agency (ANVISA)	Promotes regulations on research, production, waste disposal, and the use of nanotechnologies [155].

**Table 4:** Standards Developing Organizations (SDOs) and examples of specific standards and guidelines for nanomaterials characterisations [157].

Name of the Organisation	Example of Standards, Guidance Documents, and References
ISO Technical Committee on Nanotechnologies (TC 229)	ISO 19007:2018; In vitro MTS assay for measuring the cytotoxic effect of nanoparticles.
European Committee for Standardisation (CEN)	CEN/TC 352:WI = 00352043; Guidance on the determination of aggregation and agglomeration state of nano-objects.
American Society for Testing and Materials (ASTM), Committee E56 on Nanotechnology	ASTM E2524-08(2013); Standard test method for analysis of hemolytic properties of nanoparticles.
Canadian Standards Association (CSA)	CSA Z12885; Exposure control program for engineered nanomaterials in occupational settings.
Organization for Economic Cooperation and Development (OECD), Working Party on Manufactured Nanomaterials (WPNM)	No. 85; Evaluation of in vitro methods for human hazard assessment applied in the OECD testing program for the safety of manufactured nanomaterials.
NanoTEST, EU Seventh Framework Programme (NanoTestFP7)	Developing alternative testing strategies and high-throughput toxicity testing protocols using in vitro and in silico methods essential for the risk assessment of nanoparticles used in medical diagnostics.
